# ﻿Review of the *sequester* group of *Pteromalus* Swederus, 1795 (Hymenoptera, Pteromalidae) with description of three new species

**DOI:** 10.3897/zookeys.1251.152533

**Published:** 2025-09-04

**Authors:** Qin Li, Hannes Baur, Tongyou Zhang, Guohua Yan, Yalin Liu, Ekaterina V. Tselikh

**Affiliations:** 1 College of Life Science and Technology, Xinjiang University, 666 Shengli Road, Tianshan District, Urumqi, Xinjiang, 830046, China Xinjiang University Urumqi China; 2 Xinjiang Key Laboratory of Biological Resources and Genetic Engineering, 666 Shengli Road, Tianshan District, Urumqi, Xinjiang, 830046, China Xinjiang Key Laboratory of Biological Resources and Genetic Engineering Urumqi China; 3 Department of Invertebrates, Natural History Museum Bern (NMBE), Bernastrasse 15, 3005 Bern, Switzerland Natural History Museum Bern (NMBE) Bern Switzerland; 4 Institute of Ecology and Evolution (IEE), University of Bern, Baltzerstrasse 6, 3012 Bern, Switzerland University of Bern Bern Switzerland; 5 Zoological Institute, Russian Academy of Sciences, St Petersburg 199034, Russia Zoological Institute, Russian Academy of Sciences St Petersburg Russia

**Keywords:** Description, key, new record, new species, parasitoid, Pteromalinae, redescription, systematics, taxonomy

## Abstract

Palaearctic species of the *sequester* group of *Pteromalus* Swederus, 1795 are reviewed. Three new species, *P. boleensis* Yan, Li & Tselikh, **sp. nov.** (Kazakhstan, China), *P. longipedicellus* Yan, Li & Tselikh, **sp. nov.** (China) and *P. oscolensis* Tselikh, **sp. nov.** (Russia, China) are described and illustrated. Types of *P. cionobia* (Erdös, 1953) and *P. sequester* Walker, 1835 are redescribed. An identification key to females of the *sequester* group of *Pteromalus* is given.

## ﻿Introduction

*Pteromalus* Swederus, 1795, the largest genus in the subfamily Pteromalinae (Hymenoptera, Pteromalidae) as currently defined (Burks et al. 2022), contains 493 valid species worldwide, with the majority (371 species) described from Europe (Klimmek and Baur 2018), including 49 species from Great Britain and Ireland (Dale-Skey et al. 2016), as well as 40 species from Germany (Vidal et al. 2022). In contrast, only 15 species have been recorded from China (Yang et al. 2020; UCD Community 2025).

Due to their overall similarity, Graham (1969) included both *Pteromalus* and *Habrocytus* Thomson, 1878 into a single key to species. He organized the species into several species groups, including the *sequester* group. Later, Bouček and Graham (1978) synonymized *Habrocytus* under *Pteromalus*, and subsequent authors followed this view (Bouček and Rasplus 1991; Dale-Skey et al. 2016; Vidal et al. 2022).

Graham (1969) included five species in the *sequester* group: *P. sequester* Walker, 1835, *P. cionobia* (Erdős, 1953), *P. medicaginis* (Gahan, 1914) (was considered a synonym of *P. sequester* by Bouček (1988)), and two unidentified species “I” and “J” (unfortunately this material could not be found in the NHMUK and HDOU collections). Dzhanokmen (1998) provided a key to *Pteromalus* from Kazakhstan and also a detailed redescription of *P. sequester* (Dzhanokmen, 2001). Ye et al. (2012) redescribed *P. sequester* from China.

The aim of this work is to describe three new species of the *sequester* group of *Pteromalus*. An identification key to females of all Palaearctic species of this group is also provided.

## ﻿Materials and methods

The specimens examined in this study are deposited in the Natural History Museum (London, United Kingdom; **NHMUK**), the Hungarian Natural History Museum (Budapest, Hungary; **HNHM**), the College of Life Science and Technology, Xinjiang University (Urumqi, China; **ICXU**), the Zoological Institute of the Russian Academy of Sciences (St Petersburg, Russia; **ZISP**) and the Naturhistoriska Riksmuseet (Stockholm, Sweden; **NHRS**).

Morphological terminology, including sculpture and wing venation, follows Bouček and Rasplus (1991), Gibson (1997), and Burks et al. (2022). The flagellum consists of two anelli, six funicular segments, and the four-segmented clava. The antennal formula includes the number of segments: scapus, pedicellus, anelli, funicular segments, claval segments. The following abbreviations are used: **POL** – posterior ocellar line, the minimum distance between the posterior ocelli; **OOL** – ocello–ocular line, the minimum distance between a posterior ocellus and compound eye; **C1–C4** – claval segments; **M** – marginal vein; **S** – stigmal vein; **PM** – postmarginal vein; **F1–F6** – funicular segments; **Mt2–Mt8** – metasomal tergites (**Mt1** – petiole). The scape is measured without the radicle; the pedicel is measured in lateral view. The distance between the clypeal lower margin and the toruli is measured from the lower margins of the toruli. Eye height is measured as the maximum diameter, eye length as the minimum diameter. The mesosoma and metasoma are measured in lateral view, the latter including the ovipositor sheaths.

Specimens were examined using Nikon SMZ 745T and Olympus SZX12 stereomicroscopes. Photographs were taken with a Canon EOS 70D digital camera mounted on an Olympus SZX10 microscope (ZISP specimens), and a Nikon DS-Fi3 digital camera attached to a Nikon SMZ 25 stereomicroscope (ICXU specimens). The acquired images were then processed with Helicon Focus.

## ﻿Taxonomy

**Genus *Pteromalus* Swederus, 1795**

### ﻿*Pteromalus sequester* group

**Diagnosis.** Anterior margin of clypeus deeply incised (Figs 8, 16, 22, 30, 34). Pronotal collar medially 0.17–0.33 times as long as mesoscutum. Propodeum smooth (Figs 5, 12, 27, 33) or reticulate (Figs 18, 21); nucha narrow, alutaceous or smooth (Figs 5, 12, 18, 27, 33). Basal cell and basal vein of fore wing bare; lower surface of costal cell with hairs usually asetose in the middle (Figs 6, 11, 19, 28, 37).

**Hosts.** Primary parasitoids of coleopterans of the families Apionidae, Bruchidae, Curculionidae; dipterans of the families Cecidomyiidae and Tephritidae; hymenopterans of the family Eurytomidae; lepidopterans of the family Pyralidae (Tselikh 2019; UCD Community 2025).

**Distribution.** Nearctic, Palaearctic, Oriental, Afrotropical, Neotropical and Australian regions (Tselikh 2019; UCD Community 2025).

**Comments.***Pteromalus janssoni* (Graham, 1969) (Figs 39–43) is also close to the *sequester* group due to a deeply incised anterior margin of the clypeus (Fig. 42) and narrow nucha of propodeum (Fig. 40), but this species does not belong to this group because of clear differences presented in the key.

### ﻿Key to species of the *sequester* group of *Pteromalus* Swederus, 1795 based on females

**Table:** 

1	Basal cell of fore wing with scattered hairs; basal vein pilose; lower surface of costal cell with complete row of hairs (Fig. 43). Pronotal collar medially at most 0.1 times as long as mesoscutum (Fig. 40)	***P. janssoni* (Graham, 1969)**
–	Basal cell and basal vein of fore wing bare; lower surface of costal cell with hairs usually asetose in the middle (Figs 6, 11, 19, 28, 37). Pronotal collar medially 0.17–0.33 times as long as mesoscutum (Figs 4, 13, 18, 25, 36)	**2**
2	F1 1.28–1.57 times times as long as broad (Figs 3, 7, 35). Mt2 with sinuous hind margin (Figs 2, 38)	**3**
–	F1 1.00–1.11 times times as long as broad (Figs 15, 20, 26). Mt2 with evenly rounded hind margin (Figs 10, 21, 29), except *P. reticulatus* sp. nov. (Fig. 36)	**4**
3	Scape reaching vertex (Fig. 32). POL 1.50–1.63 times OOL. F6 as long as F5 and 1.00–1.05 times as long as broad (Fig. 35)	***P. sequester* Walker, 1835**
–	Scape reaching lower edge of median ocellus (Fig. 1). POL 1.90–2.25 times OOL. F6 shorter than F5 and 0.65–0.75 times as long as broad (Fig. 3)	***P. boleensis* sp. nov.**
4	Distance between antennal toruli and lower margin of clypeus 0.68–0.75 times distance between antennal toruli and median ocellus. Pedicel 2.22–2.36 times as long as broad and 2.16–2.22 times as long as F1 (Fig. 20). Fore wing with M 1.55–1.70 times as long as PM and 1.55–1.75 times as long as S (Fig. 19)	***P. longipedicellus* sp. nov.**
–	Distance between antennal toruli and lower margin of clypeus 0.94–1.00 times distance between antennal toruli and median ocellus. Pedicel 1.40–1.82 times as long as broad and 1.00–1.47 times as long as F1 (Figs 15, 26, 35). Fore wing with M 1.00–1.19 times as long as PM and 1.32–1.50 times as long as S (Figs 11, 28, 34)	**5**
5	Propodeal plicae incomplete (Fig. 12). Head in dorsal view 2.48–2.50 times as broad as long (Fig. 14). Pedicel 1.00–1.11 times as long as F1 (Fig. 15)	***P. cionobia* (Erdős, 1953)**
–	Propodeal plicae complete (Fig. 27). Head in dorsal view 2.24–2.30 times as broad as long (Fig. 31). Pedicel 1.40–1.47 times as long as F1 (Fig. 26)	***P. oscolensis* sp. nov.**

#### ﻿ Pteromalus boleensis

Taxon classificationAnimaliaHymenopteraPteromalidae

Yan, Li & Tselikh sp. nov.

E229A785-616D-5D10-BDC5-5B96E449CD61

https://zoobank.org/B8536B13-A2D3-4507-ADC1-DCF4996EC7AF

Figs 1–8

##### Type material.

***Holotype***: • female, China: “Xinjiang, Bole, Alashankou-Tianshan route, sweeping, 44°52'05"N, 82°2'45"E, 542 m, 26.VII.2022, coll. Qin Li group” (ICXU). ***Paratypes*** • 3 females, same labels as holotype (ICXU) • 4 females, “Bozhou, Alashankou City, Abihu Town, sweeping, 45°11'40"N, 82°34'20"E, 335 m, 24.VI.2022, coll. Qin Li group” (ICXU) • 5 females, “Bozhou, Jinghe County, sweeping, 45°11'41"N, 82°53'43"E, 267 m, 24.VI.2022, coll. Qin Li group” (ICXU) • 2 females, “Altay Prefecture, Qinghe County, sweeping, 46°55'43"N, 90°00'58"E, 1427 m, 22.VI.2021, coll. Qin Li group” (ICXU) • 1 female, “Altay Prefecture, Fuyun County, Turhong Township, sweeping, 47°01'02"N, 89°50'48"E, 1286 m, 22.VI.2021, coll. Qin Li group” (ICXU) • 3 females, “Altay Prefecture, Habahe County, Saltamu Town, sweeping, 48°00'23"N, 86°32'40"E, 440 m, 26.VI.2021, coll. Qin Li group” (ICXU) • 1 female, “Altay Prefecture, Jimunai County, sweeping, 47°44'17"N, 86°2'29"E, 562 m, 26.VI.2021, coll. Qin Li group” (ICXU) • 1 female, “Altay Prefecture, Altay City, sweeping, 47°42'41"N, 88°13'31"E, 710 m, 12.VI.2020, coll. Qin Li group” (ICXU) • 1 female, “Tacheng Prefecture, Emin County, sweeping, 46°24'17"N, 83°48'16"E, 585 m, 22 VI 2021, coll. Qin Li group” (ICXU) • 1 female, “Tacheng Prefecture, Emin County, Marelesu Town, sweeping, 46°25'55"N, 83°44'48"E, 509 m, 28.VI.2021, coll. Qin Li group” (ICXU) • 2 females, “Tiemen Pass of the Second Division of the Construction Corps, sweeping, 40°40'12"N, 87°34'48"E, 847 m, 27.VII.2022, coll. Zhulidezi Aishan group” • 1 female, “Ili Prefecture, Huocheng County, Huiyuan Town, sweeping, 43°58'00"N, 80°52'55"E, 515 m, 5.VII.2021, coll. Qin Li group” • 2 females, “Ili Prefecture, Xinyuan County, Areletobe Town, sweeping, 43°24'12"N, 83°34'53"E, 1044 m, 11.VII.2021, coll. Qin Li group” • 1 female, “Xinjiang, Kezhou, Atush City, Ahu Township, sweeping, 39°43'23"N, 76°9'04"E, 1349 m, 28.VII.2022, coll. Qin Li group” • 1 female, “Urumqi City, Shuimogou District, Boda campus of Xinjiang University, sweeping, 43°50'55"N, 87°44'35"E, 837 m, 10.VIII.2022, coll. Qin Li group” (ICXU). Kazakhstan • 16 females, “Kazakhstan, 200 km S Kustanay, Naurz. Zap., 30.VIII.1973, coll. K. Dzhanokmen” (ZISP).

##### Description.

**Female.** Body length 2.19–2.23 mm; fore wing length 1.76–1.80 mm.

***Coloration*.** Head metallic dark bluish-green with diffuse coppery lustre; antenna with scape, pedicel, and anelli yellowish-brown; F1–F6 and clava brown in dorsal view, yellowish-brown in ventral view. Mesosoma and all coxae metallic dark bluish-green with diffuse coppery lustre; all femora basally dark, apically yellowish-brown, all tibiae and tarsi yellow. Fore wing hyaline, venation yellowish-brown. Metasoma dorsally metallic dark blue and in middle part brown; ovipositor sheaths brown.

***Sculpture*.** Head reticulate; clypeus radially striate. Mesosoma reticulate; propodeum smooth. Metasoma weakly alutaceous and shiny.

***Head*.** Head in dorsal view 2.14–2.19 times as broad as long and 1.07–1.15 times as broad as mesoscutum; in frontal view 1.24–1.26 times as broad as high. POL 1.90–2.25 times as long as OOL. Eye height 1.39–1.44 times eye length and 1.85–2.15 times as long as malar space. Distance between antennal toruli and lower margin of clypeus equal to distance between antennal toruli and median ocellus. Antenna with scape 0.76–0.77 times as long as eye height and 1.06–1.11 times as long as eye length; pedicel 1.75–1.80 times as long as broad and 0.95–1.11 times F1; combined length of pedicel and flagellum 0.80–0.82 times breadth of head; F1 1.28–1.40 times as long as broad, with 2 irregular rows of sensilla; F6 0.65–0.75 times as long as broad; clava 1.78–2.05 times as long as broad, with small micropilosity area on C3 and C4. Anterior margin of clypeus deeply incised.

**Figures 1–8.**
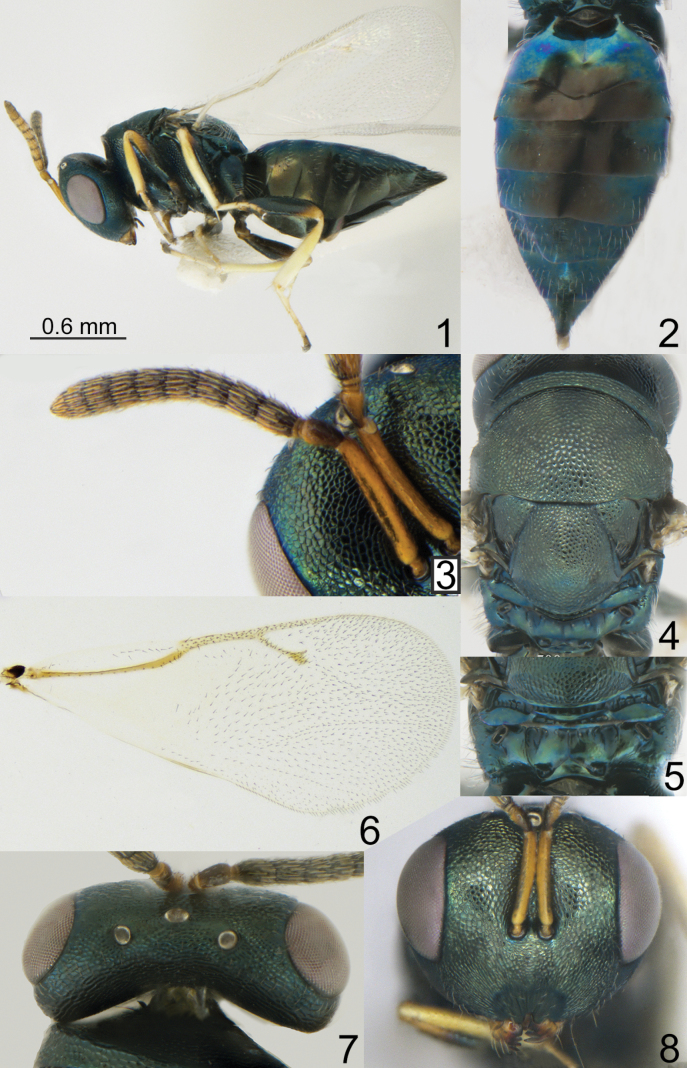
*Pteromalus boleensis* sp. nov. Yan, Li & Tselikh, female, holotype (ICXU). 1. Habitus, lateral view; 2. Metasoma, dorsal view; 3. Antenna; 4. Mesosoma, dorsal view; 5. Propodeum, dorsal view; 6. Fore wing; 7. Head, dorsal view; 8. Head, frontal view.

***Mesosoma*.** Mesosoma 1.30–1.47 times as long as broad. Scutellum moderately arched, 1.10–1.12 times as long as broad, frenal area hardly distinct. Pronotal collar medially 0.19–0.23 times as long as mesoscutum. Propodeum 0.33–0.38 times as long as scutellum; plicae and median carina complete; nucha short. Fore wing 2.22–2.28 times as long as its maximum width; basal cell and basal vein bare; speculum open below; M 1.05–1.17 times as long as PM and 1.27–1.44 times as long as S.

***Metasoma*.** Metasoma 1.73–1.92 times as long as broad, 1.40–1.62 times as long as mesosoma and 1.09–1.21 times as long as mesosoma and head. Petiole strongly transverse. Mt2 with sinuous hind margin. Ovipositor sheath projecting slightly beyond apex of metasoma.

**Male.** Unknown.

##### Etymology.

The species is named after the type locality – Bole, Xinjiang, China (adjective).

##### Distribution.

Kazakhstan, China.

##### Comments.

*Pteromalus boleensis* sp. nov. belongs to a group of species that have a long F1 with two irregular rows of sensilla and Mt2 with sinuous hind margin. This species is very similar to *P. sequester*; the differences between these species are given in the key.

#### ﻿ Pteromalus cionobia

Taxon classificationAnimaliaHymenopteraPteromalidae

(Erdös, 1953)

AB3397DE-CFD1-577D-8487-4FD8FF0586F5

Figs 9–16

Cecidostiba cionobia Erdös, 1953: 228. Lectotype female (HNHM, examined). Designated by Graham (1969: 555).

##### Type material.

***Lectotype*** • female, “Högvész 1947.VIII.23 dr. Erdös”, “Lectotypus *Cecidostiba cionobia* Erd., 1953", “Lectotypus M. de V. Graham”, “♀”, “Hym. Typ. No. 6704 Mus. Budapest” (HNHM). ***Paratype*** • 1 female, “Högvész 1947.VIII.21 dr. Erdös”, “Paralectotypus ♀ *Cecidostiba cionobia* Erd.”, “Hym. Typ. No. 6705 Mus. Budapest” (HNHM).

##### Additional material examined.

Germany • 1 female, “D. Baden-Württemberg, Grenzach-Wyhlen, Mal.-F. 4 Hornfelsen, Gebüschrand, 47°33'30"N, 07°38'48"E, 325 m, 18.VII–2.VIII.2008, coll. Doczkal, Ssymank, grf0409” (SMNS). Spain • 2 females, “ESPANGE, Gatovă, Fauchage, Martinez, 11.V.1989, coll. Rasplus” (CBGP). Russia • 1 female, “Belgorod Prov., Novooskolskii Distr., 10 km S of Novy Oskol City, “Belogorie” Reserve, “Stenki Izgor’ya”, 50°40'56"N, 37°47'16"E, 14.VIII.2020, coll. E. Tselikh” (ZISP).

##### Description.

**Female.** Body length 3.00–3.10 mm; fore wing length 2.10–2.30 mm.

**Figures 9–16.**
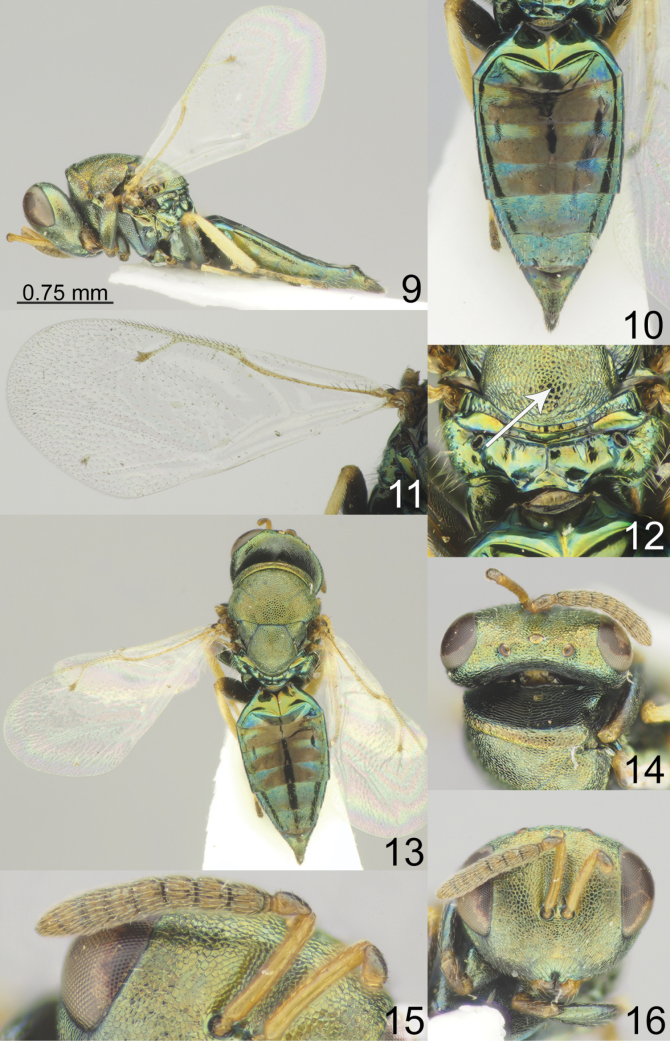
*Pteromalus cionobia* (Erdös, 1953), female, not holotype (ZISP). 9. Habitus, lateral view; 10. Metasoma, dorsal view; 11. Fore wing; 12. Propodeum, dorsal view; 13. Habitus, dorsal view; 14. Head, dorsal view; 15. Antenna; 16. Head, frontal view.

***Coloration*.** Head metallic green with diffuse coppery lustre; antenna with scape yellowish-brown; pedicel, anelli, F1-F6 and clava brown. Mesosoma, all coxae and all femora green with diffuse coppery lustre; all tibiae and tarsi yellow. Fore wing hyaline, venation yellowish-brown. Metasoma dorsally metallic bluish-green and in middle part brown; ovipositor sheaths brown.

***Sculpture*.** Head reticulate; clypeus radially striate. Mesosoma reticulate; propodeum smooth. Metasoma weakly alutaceous and shiny.

***Head*.** Head in dorsal view 2.48–2.50 times as broad as long and 1.08–1.09 times as broad as mesoscutum; in frontal view 1.27–1.29 times as broad as high. POL 1.85–2.11 times as long as OOL. Eye height 1.50 times eye length and 2.00 times as long as malar space. Distance between antennal toruli and lower margin of clypeus equal to distance between antennal toruli and median ocellus. Antenna with scape 0.78–0.80 times as long as eye height and 1.14–1.18 times as long as eye length; pedicel 1.54–1.62 times as long as broad and 1.00–1.11 times F1; combined length of pedicel and flagellum 0.79–0.80 times breadth of head; F1 1.10–1.16 times as long as broad, with 1 regular row of sensilla; F6 0.56–0.65 times as long as broad; clava 1.42–1.44 times as long as broad, with small micropilosity area on C3 and C4. Anterior margin of clypeus deeply incised.

***Mesosoma*.** Mesosoma 1.35–1.40 times as long as broad. Scutellum moderately arched, 1.03–1.08 times as long as broad, frenal area hardly distinct. Pronotal collar medially 0.16–0.18 times as long as mesoscutum. Propodeum 0.30–0.41 times as long as scutellum; plicae not complete, median carina complete; nucha short. Fore wing 2.21–2.3 times as long as its maximum width; basal cell and basal vein bare; speculum partly open below; M 1.16–1.17 times as long as PM and 1.37–1.48 times as long as S.

***Metasoma*.** Metasoma 2.06–2.13 times as long as broad, 1.58–1.65 times as long as mesosoma and 1.16–1.17 times as long as mesosoma and head. Petiole strongly transverse. Mt2 with evenly rounded hind margin. Ovipositor sheath projecting slightly beyond apex of metasoma.

##### Distribution.

Europe, Turkey, Russia (Tselikh 2021; UCD Community 2025).

##### Biology.

Primary parasitoid of *Cionus thapsus* (Fabricius) (Coleoptera, Curculionidae) (UCD Community 2025).

##### Comments.

*Pteromalus cionobia* (Erdös, 1953) belongs to a group of species that have a short F1 with 1 regular row of sensilla and Mt2 with evenly rounded hind margin. This species is very similar to *P. oscolensis* sp. nov. and *P. reticulatus* sp. nov.; the differences between these species are given in the key.

#### ﻿ Pteromalus longipedicellus

Taxon classificationAnimaliaHymenopteraPteromalidae

Yan, Li & Tselikh sp. nov.

41A0588B-791A-5F1F-A50A-6231C9B52A3C

https://zoobank.org/1D0B464A-7A9D-447D-9557-D164CEF2BF13

Figs 17–24

##### Type material.

***Holotype*** • female, China: “Xinjiang, Emin County, sweeping, 46°21'43.91"N, 83°53'17.52"E, 645 m, 28.VI.2021, coll. Q. Li group” (ICXU). ***Paratypes*** • 2 females, “Xinjiang, Ili Prefecture, Tekes County, 43°9'19.98"N, 81°47'23.76"E, 1184 m, 9.VII.2021, coll. Qin Li group” (ICXU) • 1 female, “Altay Prefecture, Fuyun County, 47°0'53.64"N, 89°32'4.90"E, 1360 m, 11.VII.2020, coll. Qin Li group” (ICXU).

##### Description.

**Female.** Body length 2.23–2.42 mm; fore wing length1.75–1.80 mm.

**Figures 17–24.**
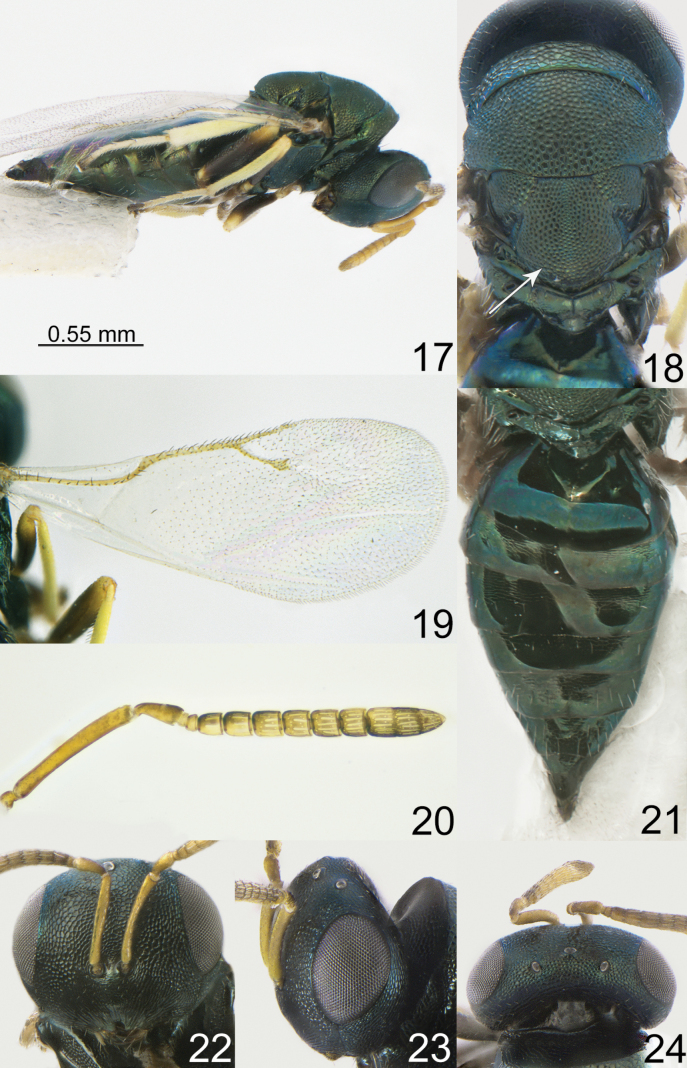
*Pteromalus longipedicellus* sp. nov. Yan, Li & Tselikh, female, holotype (ICXU). 17. Habitus, lateral view; 18. Mesosoma, dorsal view; 19. Fore wing; 20. Antenna; 21. Metasoma, dorsal view; 22. Head, frontal view; 23. Head, lateral view; 24. Head, dorsal view.

***Coloration*.** Head metallic dark bluish-green with diffuse coppery lustre; antenna with scape, pedicel, anelli, F1–F6 and clava yellowish-brown. Mesosoma and all coxae metallic dark bluish-green with diffuse coppery lustre; all femora basally dark, apically yellowish-brown, all tibiae and tarsi yellow. Fore wing hyaline, venation yellowish-brown. Metasoma dorsally metallic dark blue and in middle part brown; ovipositor sheaths brown.

***Sculpture*.** Head reticulate; clypeus radially striate. Mesosoma reticulate; propodeum weakly reticulate. Metasoma weakly alutaceous and shiny.

***Head*.** Head in dorsal view 2.00–2.09 times as broad as long and 1.09–1.16 times as broad as mesoscutum; in frontal view 1.23–1.26 times as broad as high. POL 2.00–2.20 times as long as OOL. Eye height 1.44–1.45 times eye length and 1.76–1.82 times as long as malar space. Distance between antennal toruli and lower margin of clypeus 0.68–0.75 times distance between antennal toruli and median ocellus. Antenna with scape 0.80–0.90 times as long as eye height and 1.30–1.35 times as long as eye length; pedicel 2.22–2.36 times as long as broad and 2.16–2.22 times F1; combined length of pedicel and flagellum 0.80–0.82 times breadth of head; F1 1.00 times as long as broad, with 1 regular row of sensilla; F6 0.71–0.76 times as long as broad; clava 2.50–2.56 times as long as broad, with small micropilosity area on C3 and C4. Anterior margin of clypeus deeply incised.

***Mesosoma*.** Mesosoma 1.43–1.50 times as long as broad. Scutellum moderately arched, as long as broad, frenal area hardly distinct. Pronotal collar medially 0.28–0.33 times as long as mesoscutum. Propodeum 0.36–0.47 times as long as scutellum; plicae and median carina complete; nucha short. Fore wing 2.15–2.32 times as long as its maximum width; basal cell and basal vein bare; speculum open below; M 1.55–1.70 times as long as PM and 1.55–1.75 times as long as S.

***Metasoma*.** Metasoma 1.81–2.00 times as long as broad, 1.26–1.36 times as long as mesosoma and 1.00–1.05 times as long as mesosoma and head. Petiole strongly transverse. Mt2 with evenly rounded hind margin. Ovipositor sheath projecting slightly beyond apex of metasoma.

**Male.** Unknown.

##### Etymology.

From the Latin “longus” and “pedicellus”, referring to the long pedicel of this species.

##### Distribution.

China.

##### Comments.

*Pteromalus longipedicellus* sp. nov. belongs to a group of species that have a short F1 with 1 regular row of sensilla and Mt2 with evenly rounded hind margin. This species is similar to *P. cionobia* (Erdős, 1953), *P. oscolensis* sp. nov., and *P. reticulatus* sp. nov.; the differences between these species are given in the key.

#### ﻿ Pteromalus oscolensis

Taxon classificationAnimaliaHymenopteraPteromalidae

Tselikh sp. nov.

A7949268-447F-5475-B43C-2B3D977BE80D

https://zoobank.org/997C6E6F-264E-4CE8-95B2-7B52861640E6

Figs 25–31

##### Type material.

***Holotype*** • female, Russia: “Belgorod Prov., 10 km S of Novy Oskol City, “Belogorie” Reserve, “Stenki Izgor’ya”, 50°40'56"N, 37°47'16"E, 14.VIII.2020, coll. E. Tselikh” (ZISP). ***Paratypes*** • 7 females, Russia • same label as holotype • 2 females, “Roven’ki Vill., “Roven’ki Nature Park,” “Aydar,” 49°50'21"N, 39°19'15"E, 12.VIII.2020, coll. E. Tselikh” (ZISP) • 4 females, “Voronezh Prov., 20 km SW of Rossosh’ City, Zhilino Vill., 49°49'58"N, 39°19'48"E, 10–11.VIII.2020, coll. O. Kosheleva and E. Tselikh” (ZISP). China • 2 females, “Urumqi City, Shuimogou District, Boda campus of Xinjiang University, sweeping, 87°44'35"E, 43°50'55"N, 837 m, 10.VIII.2022, coll. Qin Li group” (ICXU) • 1 female, “Bozhou, Alashankou City, Abihu Town, sweeping, 82°34'20"E, 45°11'40"N, 335 m, 24.VI.2022, coll. Qin Li group” (ICXU).

**Figures 25–31.**
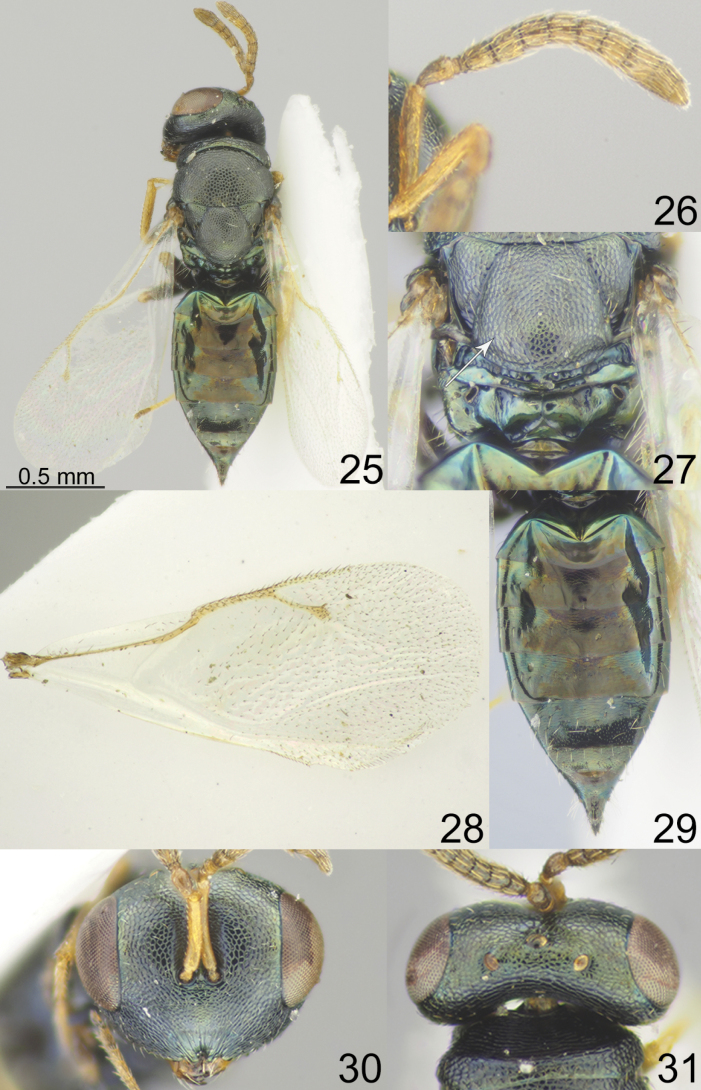
*Pteromalus oscolensis* sp. nov. Tselikh, female, holotype (ZISP). 25. Habitus, dorsal view; 26. Antenna; 27. Propodeum, dorsal view; 28. Fore wing; 29. Metasoma, dorsal view; 30. Head, frontal view; 31. Head, dorsal view.

##### Description.

**Female.** Body length 1.85–2.00 mm; fore wing length 1.45–1.50 mm.

***Coloration*.** Head metallic dark bluish-green with diffuse coppery lustre; antenna with scape, pedicel, anelli, F1–F6 and clava yellowish-brown. Mesosoma and all coxae metallic dark bluish-green with diffuse coppery lustre; all femora basally dark, apically yellowish-brown, all tibiae and tarsi yellow. Fore wing hyaline, venation yellowish-brown. Metasoma dorsally metallic dark blue and in middle part brown; ovipositor sheaths brown.

***Sculpture*.** Head reticulate; clypeus radially striate. Mesosoma reticulate; propodeum smooth. Metasoma weakly alutaceous and shiny.

***Head*.** Head in dorsal view 2.24–2.30 times as broad as long and 1.21–1.22 times as broad as mesoscutum; in frontal view 1.22–1.25 times as broad as high. POL 1.78–1.82 times as long as OOL. Eye height 1.38–1.40 times eye length and 2.00–2.09 times as long as malar space. Distance between antennal toruli and lower margin of clypeus 0.94–1.00 times distance between antennal toruli and median ocellus. Antenna with scape 0.72–0.75 times as long as eye height and 1.00 times as long as eye length; pedicel 1.75–1.82 times as long as broad and 1.40–1.47 times F1; combined length of pedicel and flagellum 0.87–0.88 times breadth of head; F1 1.10–1.11 times as long as broad, with 1 regular row of sensilla; F6 0.88–0.90 times as long as broad; clava 1.80–2.00 times as long as broad, with small micropilosity area on C3 and C4. Anterior margin of clypeus deeply incised.

***Mesosoma*.** Mesosoma 1.42–1.43 times as long as broad. Scutellum moderately arched, 0.93–0.97 times as long as broad, frenal area hardly distinct. Pronotal collar medially 0.21–0.23 times as long as mesoscutum. Propodeum 0.37–0.39 times as long as scutellum; plicae and median carina complete; nucha short. Fore wing 2.28–2.30 times as long as its maximum width; basal cell and basal vein bare; speculum open below; M 1.00–1.19 times as long as PM and 1.32–1.47 times as long as S.

***Metasoma*.** Metasoma 1.89–1.97 times as long as broad, 1.37–1.42 times as long as mesosoma and 0.93–1.06 times as long as mesosoma and head. Petiole strongly transverse. Mt2 with evenly rounded hind margin. Ovipositor sheath projecting slightly beyond apex of metasoma.

**Male.** Unknown.

##### Etymology.

The species is named after the type locality, Novy Oskol City (adjective).

##### Distribution.

Russia, China.

##### Comments.

*Pteromalus oscolensis* sp. nov. belongs to a group of species that have a short F1 with one regular row of sensilla and Mt2 with evenly rounded hind margin. This species is very similar to *P. cionobia* (Erdős, 1953); the differences between these species are given in the key.

#### ﻿ Pteromalus sequester

Taxon classificationAnimaliaHymenopteraPteromalidae

Walker, 1835

6EE9223B-BE18-5137-AF02-EE28CB4F3782

Figs 32–39

##### Synonymy.

For synonymy, see UCD Community (2025).

##### Type material.

***Lectotype*** • female, Great Britain: “LECTOTYPE”, “Stood under name in old B. M. Coll. C. Waterhouse”, “*Pteromalus sequester* Walker”, “in genus *Trichomalus* Ch. Ferriere det.”, “*Pteromalus sequester* Walker LECTOTYPE M. de V. Graham det. 1968", “B.M. TYPE HYM 5.2754", “NHMUK 013457284" (NHMUK).

##### Additional material examined.

Russia • females, “Krasnodar Terr., Sochi, Lazarevskoe, 3–6.VI.1974, coll. V. Tobias” (ZISP) • 1 female, “Smolensk Prov., near Smolensk City, 54°49'01"N, 32°04'50"E, 22.VIII.2020, coll. O. Kosheleva” (ZISP) • 1 female, “Primorskii Terr., Vladivostok, Akademgorodok, 27.VII.1961 coll. V. Trjapitzin” (ZISP) • 2 females, “Vladivostok, Sedanka, 4.VIII.2001, coll S. Belokobylskij” (ZISP).

##### Description.

**Female.** Body length 2.80–3.10 mm; fore wing length 2.30–2.50 mm.

***Coloration*.** Head metallic green with diffuse coppery lustre; antenna with scape yellowish-brown; pedicel, anelli, F1–F6 and clava brown in dorsal view, yellowish-brown in ventral view. Mesosoma, all coxae and all femora metallic green with diffuse coppery lustre; all tibiae and tarsi yellow. Fore wing hyaline, venation yellowish-brown. Metasoma dorsally metallic green with diffuse coppery lustre and in middle part brown with diffuse violet lustre; ovipositor sheaths brown.

***Sculpture*.** Head reticulate; clypeus radially striate. Mesosoma reticulate; propodeum smooth. Metasoma weakly alutaceous and shiny.

***Head*.** Head in dorsal view 2.21–2.45 times as broad as long and 1.12–1.19 times as broad as mesoscutum; in frontal view 1.23–1.26 times as broad as high. POL 1.50–1.63 times as long as OOL. Eye height 1.40–1.47 times eye length and 1.85–2.20 times as long as malar space. Distance between antennal toruli and lower margin of clypeus 1.00–1.17 times distance between antennal toruli and median ocellus. Antenna with scape 0.60–0.80 times as long as eye height and 1.00–1.21 times as long as eye length; pedicel 1.67–1.77 times as long as broad and 0.86–1.10 times F1; combined length of pedicel and flagellum 0.84–0.89 times breadth of head; F1 1.44–1.57 times as long as broad, with 2 irregular rows of sensilla; F6 1.00–1.05 times as long as broad; clava 1.95–2.12 times as long as broad, with small micropilosity area on C3 and C4. Anterior margin of clypeus deeply incised.

***Mesosoma*.** Mesosoma 1.34–1.46 times as long as broad. Scutellum moderately arched, 0.92–1.05 times as long as broad, frenal area hardly distinct. Pronotal collar medially 0.17–0.22 times as long as mesoscutum. Propodeum 0.40–0.48 times as long as scutellum; plicae and median carina complete; nucha short. Fore wing 2.12–2.30 times as long as its maximum width; basal cell and basal vein bare; speculum open below; M 1.08–1.26 times as long as PM and 1.39–1.51 times as long as S.

***Metasoma*.** Metasoma 1.65–1.85 times as long as broad, 1.24–1.29 times as long as mesosoma and 1.00 times as long as mesosoma and head. Petiole strongly transverse. Mt2 with evenly curved hind margin. Ovipositor sheath projecting slightly beyond apex of metasoma.

**Figures 32–39.**
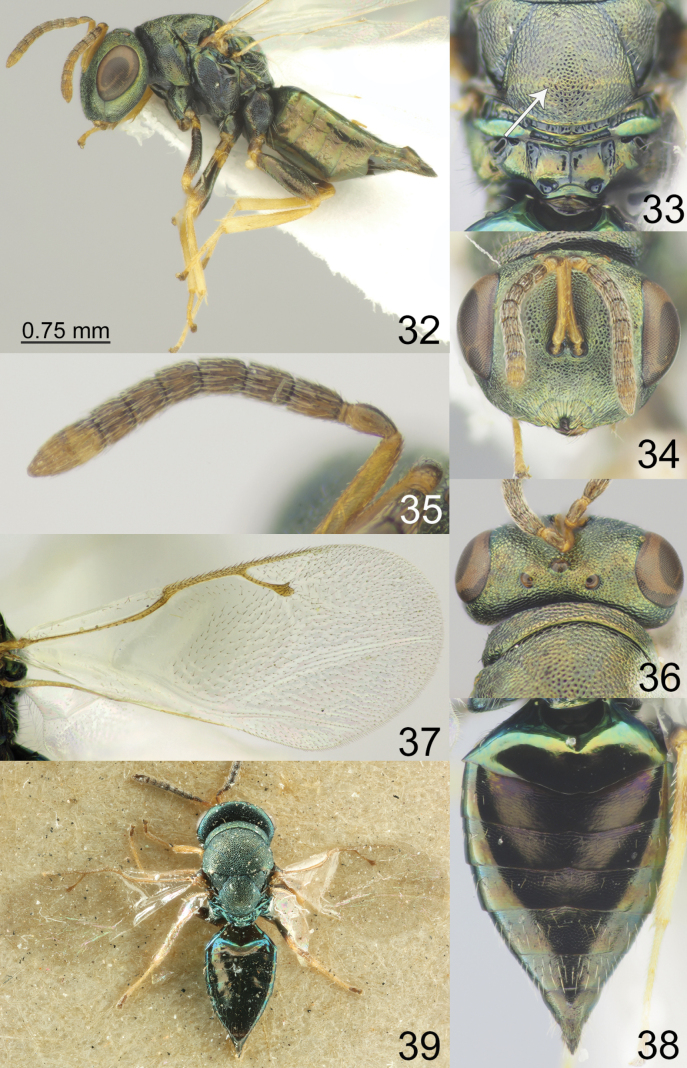
*Pteromalus sequester* Walker, 1835, female, not type (ZISP). 39. Female, lectotype (NHMUK); 32. Habitus, lateral view; 33. Propodeum, dorsal view; 34. Head, frontal view; 35. Antenna; 36. Head, dorsal view; 37. Fore wing; 38. Metasoma, dorsal view; 39. Habitus, dorsal view.

**Figures 40–44.**
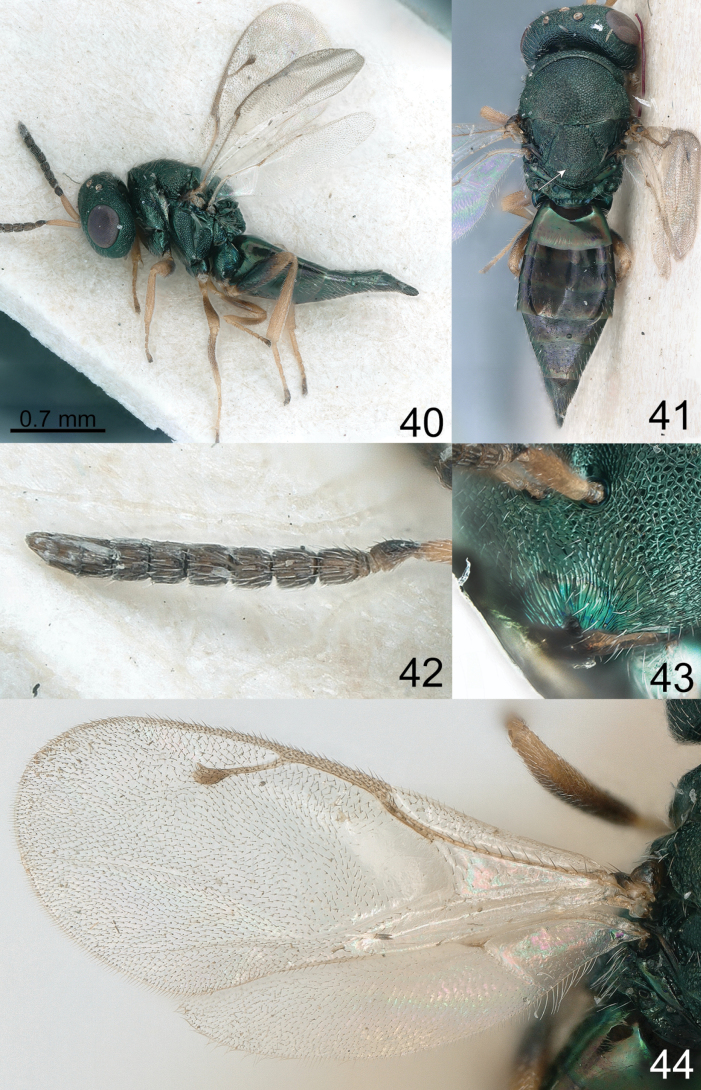
*Pteromalus janssoni* (Graham, 1969), female, not type (NHRS). 40. Habitus, lateral view; 41. Habitus, dorsal view; 42. Antenna; 43. Clypeus; 44. Wings.

##### Distribution.

Australia, Europe (including Canary Islands), India, Iran, Iraq, Israel, Kazakhstan, Mexico, New Zealand, Russia, Turkey, USA (UCD Community 2025).

##### Biology.

Primary parasitoid of coleopterans of the families Bruchidae, Apionidae and Curculionidae, dipterans of the families Cecidomyiidae and Tephritidae, hymenopterans of the family Eurytomidae and lepidopterans of the family Pyralidae (UCD Community 2025).

##### Comments.

*Pteromalus sequester* Walker, 1835 belongs to a group of species that have a long F1 with two irregular rows of sensilla and Mt2 with evenly curved hind margin. This species is very similar to *P. boleensis* sp. nov.; the differences between these species are given in the key.

## Supplementary Material

XML Treatment for Pteromalus boleensis

XML Treatment for Pteromalus cionobia

XML Treatment for Pteromalus longipedicellus

XML Treatment for Pteromalus oscolensis

XML Treatment for Pteromalus sequester

## ﻿Additional information

### Use of AI

No use of AI was reported.

### Author ORCIDs

Qin Li https://orcid.org/0000-0002-1780-7332

Hannes Baur https://orcid.org/0000-0003-1360-3487

Tongyou Zhang https://orcid.org/0009-0000-5425-3765

Yalin Liu https://orcid.org/0009-0007-6558-267X

Ekaterina V. Tselikh https://orcid.org/0000-0002-9184-043X

### Data availability

All of the data that support the findings of this study are available in the main text.
